# Out-of-Distribution Detection Algorithms for Robust Insect Classification

**DOI:** 10.34133/plantphenomics.0170

**Published:** 2024-04-30

**Authors:** Mojdeh Saadati, Aditya Balu, Shivani Chiranjeevi, Talukder Zaki Jubery, Asheesh K. Singh, Soumik Sarkar, Arti Singh, Baskar Ganapathysubramanian

**Affiliations:** ^1^ Department of Computer Science, Iowa State University, Ames, IA, USA.; ^2^ Department of Mechanical Engineering, Iowa State University, Ames, IA, USA.; ^3^ Department of Agronomy, Iowa State University, Ames, IA, USA.

## Abstract

Plants encounter a variety of beneficial and harmful insects during their growth cycle. Accurate identification (i.e., detecting insects’ presence) and classification (i.e., determining the type or class) of these insect species is critical for implementing prompt and suitable mitigation strategies. Such timely actions carry substantial economic and environmental implications. Deep learning-based approaches have produced models with good insect classification accuracy. Researchers aim to implement identification and classification models in agriculture, facing challenges when input images markedly deviate from the training distribution (e.g., images like vehicles, humans, or a blurred image or insect class that is not yet trained on). Out-of-distribution (OOD) detection algorithms provide an exciting avenue to overcome these challenges as they ensure that a model abstains from making incorrect classification predictions on images that belong to non-insect and/or untrained insect classes. As far as we know, no prior in-depth exploration has been conducted on the role of the OOD detection algorithms in addressing agricultural issues. Here, we generate and evaluate the performance of state-of-the-art OOD algorithms on insect detection classifiers. These algorithms represent a diversity of methods for addressing an OOD problem. Specifically, we focus on extrusive algorithms, i.e., algorithms that wrap around a well-trained classifier without the need for additional co-training. We compared three OOD detection algorithms: (a) maximum softmax probability, which uses the softmax value as a confidence score; (b) Mahalanobis distance (MAH)-based algorithm, which uses a generative classification approach; and (c) energy-based algorithm, which maps the input data to a scalar value, called energy. We performed an extensive series of evaluations of these OOD algorithms across three performance axes: (a) Base model accuracy: How does the accuracy of the classifier impact OOD performance? (b) How does the level of dissimilarity to the domain impact OOD performance? (c) Data imbalance: How sensitive is OOD performance to the imbalance in per-class sample size? Evaluating OOD algorithms across these performance axes provides practical guidelines to ensure the robust performance of well-trained models in the wild, which is a key consideration for agricultural applications. Based on this analysis, we proposed the most effective OOD algorithm as wrapper for the insect classifier with highest accuracy. We presented the results of its OOD detection performance in the paper. Our results indicate that OOD detection algorithms can significantly enhance user trust in insect pest classification by abstaining classification under uncertain conditions.

## Introduction

Insect pest infestation can be observed at all stages of growth in crop plants, negatively affecting the quality and quantity of yields in agriculture [1]. Accurate detection of insects is imperative for prompt, timely, and optimal decision-making [2]. For instance, early detection of pest infestations can allow farmers to take timely and appropriate action to prevent or minimize crop damage [3,4]. More importantly, accurate detection allows farmers to identify the specific pest species that are causing damage [5], enabling them to use targeted pest control methods instead of blanket applications of pesticides. This reduces the risk of harm to beneficial insects and other non-target organisms. Furthermore, accurate identification of insect pests can result in effective pest control measures, which reduce the number of crop losses due to insect pests, increase the profitability of farmer operations, and reduce the amount of chemical runoff into water bodies. Finally, accurate and timely insect pest detection is also important for compliance with regulatory requirements related to pesticide use and environmental protection.

Traditionally, insect pest identification (and quantification) [6] has been performed by human experts and scouts. In the past decade, machine learning (ML) approaches have increasingly been used for automating plant stress identification, classification, and quantification [7,8]. Researchers initially used classical ML algorithms for insect identification and classification [9,10]. However, more recently, deep learning approaches have been suggested [11,12,13]. In the classical approaches, features such as color and texture are first extracted from images. Then, a classifier learns a mapping from the feature space to its corresponding label. For example, support vector machines (SVMs) have been commonly used for plant stress detection problems [14,15]. However, such approaches require extensive domain knowledge and tedious approaches for feature extraction. In the past decade, researchers have utilized deep learning approaches to perform end-to-end classification without requiring manual feature extraction. In classifying 13 soybean pests, Tetila et al. [16] compared the performance of five models (Inception-v3 [17], ResNet50 [18], VGG16 [19], VGG19 [19], and Xception [20]) across a dataset containing 5,000 samples. Li et al. [21] leveraged the GoogLeNet architecture and achieved 98% accuracy in a 10-class insect classification task using a dataset of around 7,000 images. Manual labeling of this moderately sized dataset required extensive effort by domain experts. The need for manual annotation of large datasets for training deep learning algorithms has become a major bottleneck to creating high-accuracy classifiers. Recent efforts to utilize self-supervised algorithms seek to eliminate the need for large, annotated datasets. Using self-supervised approaches, one can eliminate the need for labeling by utilizing specific data features to design pretext tasks. The model learns the underlying features and relationships between the input data by performing these pretext tasks [22,23]. In a recent study by Kar et al. [13], the authors utilized the Bootstrap Your Own Latent, a self-supervised method to create a pest classifier algorithm. This insect classifier achieved an accuracy of 93% using approximately 15,000 images spanning 22 classes of agriculturally relevant insects found in the US Midwest. A review of this rapidly growing field highlights a trend toward utilizing larger datasets such as the iNaturalist dataset (https://github.com/visipedia/inat_comp/tree/master/2021) (with >10 million images), along with weakly and self-supervised approaches to develop highly accurate classifiers for a wide range of agriculturally relevant applications. This progress opens up the possibility for practical deployment in agricultural decision support systems.

**Fig. 1.**
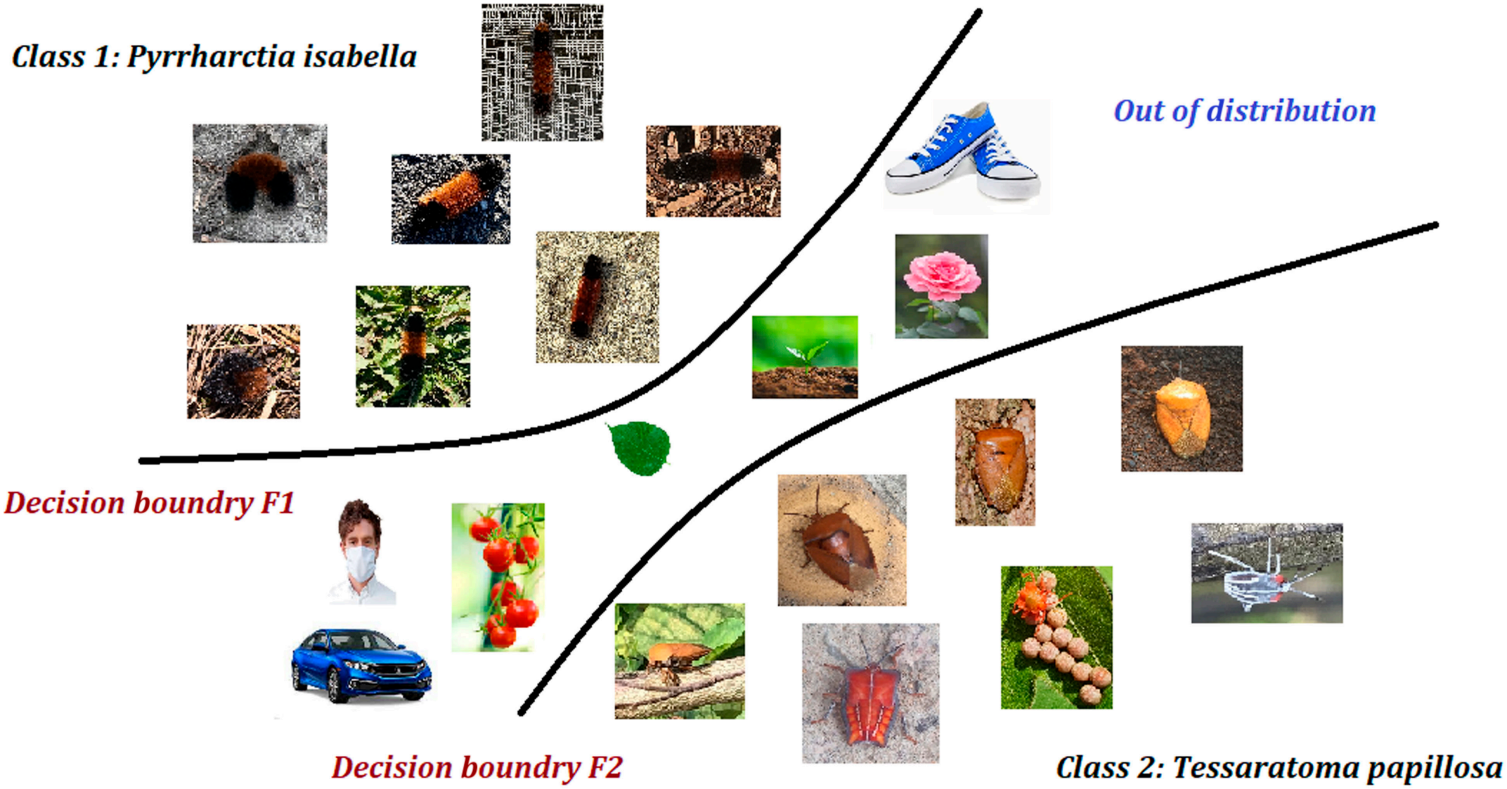
Out-of-distribution visualization in insect classification. The OOD classifier tries to define decision boundaries for each insect while ensuring no non-insect images fall into any known insect classes. There is a spectrum of OOD images. One can distinguish them as contextually near OOD (for instance, leaf images) and contextually far OOD (for instance, car image) based on their similarity to the ID images.

However, the trustworthiness of these classifiers is a potential bottleneck for their practical deployment in agricultural fields. For instance, while classifiers (or well-trained ML models in general) can achieve high classification accuracy, these models do not provide any measure of (un)certainty in making predictions or abstaining from classification in the case of uncertainty. This is particularly important for autonomous agricultural applications, where inaccurate predictions may lead to wastage of resources at the very least and substantial yield losses in the worst-case scenario. In the context of ML models for insect classification, practical deployment requires distinguishing—or at least abstaining from making predictions—when confronted with confusing or unseen images. This can occur when the model presents images of insect species that fall outside the categories it has been trained in. Such misclassification could have disastrous consequences if the model mistakenly identifies an unseen insect class, for instance, an invasive species of insect, as a harmless one. This may result in incorrect crop management decisions. Although the sizes of such confusing classes may vary, the loss of scale perspective in the image may contribute to these erroneous predictions. Thus, there is a pressing need to integrate advances in model accuracy with enhanced trustworthiness to ensure robust deployment in the field (trustworthiness can be defined in several ways [24–26]). Increasing the trustworthiness of ML models is an active area of research with several promising approaches [27–29]. One approach is to identify images that do not belong to the data distribution the model originally trained on. Such out-of-distribution (OOD) approaches can ensure that the model can avoid making predictions when facing an image that does not belong to any of the classes it is trained to predict. OOD detection has been successfully used in several applications, like autonomous systems [30,31], medical diagnosis [32–34], robotics [35,36], and social science [37], to improve the reliability and safety of systems. OOD detection is desirable in insect pest detection as it allows human intervention in case of uncertain model predictions. Figure 1 illustrates how such OOD detection can be used for the insect classification task.

For deep learning models, one of the earliest algorithms proposed for identifying OOD data is maximum softmax probability (MSP) [38]. This algorithm relies on the assumption that deep learning models are more confident in the classification of in-distribution (ID) data rather than OOD data. The algorithm uses the softmax value as a metric to measure the confidence of predictions. Due to its simplicity and good performance, this algorithm has been prevalent in addressing OOD detection. However, it has been shown in practice that MSP can produce a high false-positive rate (FPR) during OOD detection. Recently, Liu [39] showed (using both theory and empirical evidence) that an energy-based model (EBM) can be a great substitute for MSP as it is aligned with the probability density of ID data. The basic idea here is to design a function that maps the input (or input features) to a number, often called energy. This energy function is designed such that ID samples have low energy, while OOD samples exhibit higher energy. OOD boils down to comparing the energy of an unseen input against a threshold.

In contrast to these discriminative OOD algorithms, which try to find the best decision boundaries, there are also OOD algorithms based on generative models that focus on estimating the ID density [40–43]. Among generative model-based OOD algorithms, OOD detection based on MAH is the most popular, with significant recent work. Denouden [44] solves the OOD problem in the context of auto-encoder architecture, using the observation that an auto-encoder is ineffective in encoding and reconstructing OOD data in comparison to ID ones. They distinguish OOD data from ID by defining a threshold based on the MAH metric on reconstruction error. Lee [45] extracted class conditional Gaussian distributions of deep learning features based on Gaussian discriminant analysis, leading to a MAH-based confidence score. Ren et al. [46] commented that the latter algorithm suffered from near-OOD detection and offered an adjustment to the previous algorithm.

All the algorithms described above are extrusive; i.e., they wrap around a trained ML classifier without the need for additional (re)training of the classifier. Another class of OOD algorithms exists, which work by introducing part of the OOD data during the classifier training process. These intrusive approaches require co-training of the OOD detection along with the classifier. For instance, Hendrycks et al. [47] modify the classifier’s cross-entropy loss function and add an extra term to handle OOD data so that the softmax distribution for OOD data is uniform. Researchers also incorporated OOD detection into the classifier’s architecture and designed a hierarchical outlier detection algorithm (HOD) to identify the outlier dermatological conditions [48]. Fort et al. [49] and Ren et al. [42] also adjust the classifier by adding an extra class to classify the OOD data during the prediction.

We choose to focus on the extrusive algorithms from the agricultural application point of view for the following reasons: (a) the increasing availability of well-trained models for various agricultural phenotyping tasks. These models are often trained on huge datasets using significant computational effort. Retraining them to incorporate OOD detection using intrusive algorithms may be computationally infeasible for many practitioners. (b) Additionally, extrusive algorithms allow efficient personalization of the OOD detection for the specific application cases involved. That is, end-users can identify application-specific types of OOD images that are most relevant to their application.

In this paper, we explored the utility of OOD models for agricultural applications, mainly insect pest classification. This work addresses a gap to enhance the trustworthiness of artificial intelligence in the agricultural domain by tackling the uncertainty, more specifically out-of-domainness, of their decision-making. Despite the importance of the matter, little research has been done in this area. To the best of our knowledge, our work is the first scientific research conducting comprehensive analyses to compare and evaluate the performance of the most prominent OOD algorithms in an agricultural domain. As a result of our experiment, we proposed an equipped insect classifier with a high-accuracy OOD detection feature. While the experiment mainly focuses on insect classification tasks, the result of the analysis can easily be expanded to other plant science problems. We perform OOD analysis on an insect classifier that can distinguish between 142 agriculturally relevant insect pest classes. Our key contributions are as follows:

• We incorporate the concept of OOD detection for insect pest classification, using a classifier that can distinguish between 142 agriculturally relevant insect pest species trained on a large insect dataset (2 million images). This differs from previous works, which conduct their analysis on benchmark datasets such as CIFAR10 [50], CIFAR100 [50], and SVHN [51], which have relatively smaller data sizes (≤100,000 samples).

• We computationally explore the performance of three OOD methods (MSP, MAH, and EBM) for insect pest classification. We report on OOD performance using different classifier architectures, different training dataset distributions, and testing on different OOD datasets.

The paper is structured as follows. The “Materials and Methods” section provides an overview of the problem definition, including a discussion of the OOD detection methods, ID and OOD datasets, and classifier architectures. The “Results and Discussion” section details the analyses conducted to evaluate OOD detection algorithms through different performance axes. Finally, in the “Conclusions” section, concluding remarks are provided, along with a discussion of future works.

## Materials and Methods

**Fig. 2.**
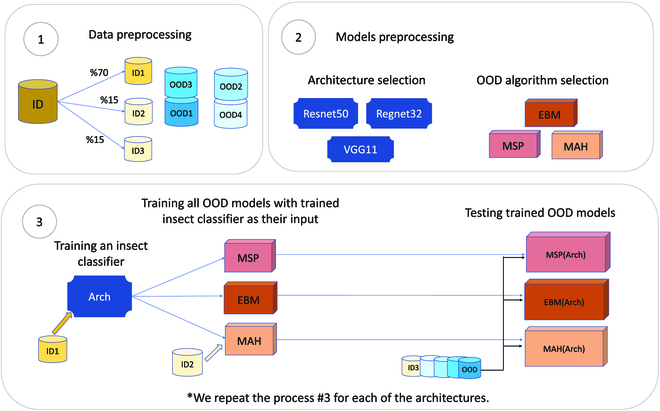
The analysis process: Box 1 represents the data. We split the ID distribution data into three sub-datasets. The first subset is used to train the insect classifiers used in this work, the second is used to train the Mahalanobis distance OOD algorithm, and the third is used to test the results. This allows strict separation of datasets for testing and evaluation. We also utilize four different OOD datasets for testing our results. Box 2 shows the three insect classifier architectures and the three OOD algorithms used. Box 3 illustrates the workflow. The training data (ID1) are used to train the insect classifier. The OOD algorithms (and ID2 data, if needed) are next trained. Finally, the test data (ID3, OOD1, OOD2, OOD3, and OOD4) are used to test the OOD performance.

To understand the characteristics of OOD algorithms in the context of insect pest classification, we selected three OOD algorithms: MSP, MAH, and EBM. MSP serves as a suitable baseline for comparison, while the MAH-based and energy-based algorithms each represent distinct families of OOD algorithms, discriminative and generative, respectively. We chose extrusive OOD algorithms as they can be applied to any arbitrary insect classifier as a wrapper. For the performance evaluation of these OOD detection algorithms and finding the most suitable OOD detection in insect pest classification tasks, we created a pipeline (Fig. 2) for comparison between OOD detection algorithms. The pipeline encompasses three main sections: data preprocessing, insect classifier training, and OOD algorithm testing. In data preprocessing, the ID data are split into three parts: ID1, ID2, and ID3. ID1 is used for training insect pest classifiers; ID2 is used for training our generative algorithm, MAH; and ID3 is used for testing OOD detection algorithm performance. To compare the OOD algorithm’s performance on different degrees of similarity between ID data and OOD data, we test them on four OOD datasets. Also, to ensure the OOD performance evaluation is independent of network architecture, we repeat the experiment on three different network architecture insect classifiers. For OOD performance evaluation, first, the OOD algorithm is wrapped around a chosen insect classifier. Then, MAH, the generative OOD algorithm, is trained on an extra ID2 validation set. After the OOD binary classifier is created, we test it to distinguish ID3 data from each of the OOD datasets. Each OOD algorithm proposes its own confidence metric that represents how confident the classifier is in its decision and, in case of low confidence or high uncertainty, reports the input as OOD. To create a binary classifier out of the OOD algorithm, we need to find a threshold on the confidence score. We find the best threshold according to the ROC curve. A threshold with the minimum false positive and highest true positive is chosen. The area under the ROC curve and true positive when false positive is 95% are reported as the performance of the OOD detection algorithm.

### OOD methods

**Fig. 3.**
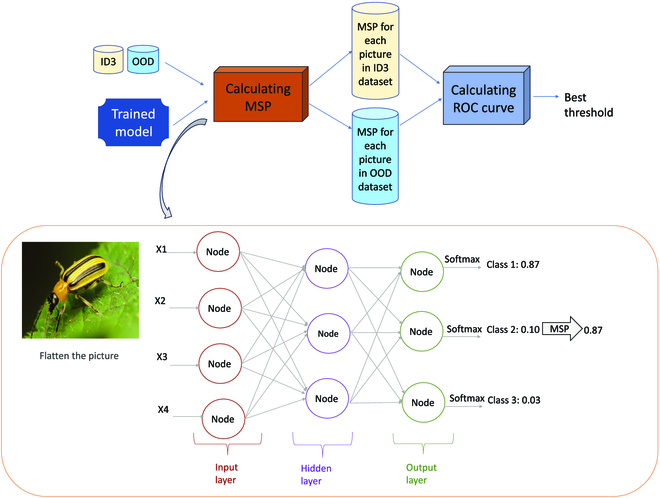
OOD detection process on MSP. For each image in the ID3 and OOD datasets, the MSP is calculated as a confidence score. Then ROC curve was plotted for all possible thresholds. The threshold with minimum false positive and maximum true positive is returned as the best threshold.

OOD detection refers to identifying whether a given input data point belongs to the same distribution as the training data. In other words, it aims to identify when a model encounters data significantly different from what it has been trained on. We detail the three OOD algorithms considered:

**•** MSP [38] is a simple yet effective method for OOD detection in deep learning models. It is based on the fact that softmax output probabilities (the last layer of the deep learning network before classification) for ID data tend to be more confident than those for OOD data. Specifically, the MSP method computes the maximum value of the softmax probabilities for a given input data point and compares it to a threshold value. If the maximum value is below the threshold, the data point is considered OOD. The threshold is usually determined based on a validation set or using a predefined value (Fig. 3).

MSP has been shown to perform well in detecting OOD data in various applications, such as image classification, natural language processing, and speech recognition [38]. It is also computationally efficient and easy to implement as it only uses softmax value extracted from the last layer of the neural network, making it a popular choice for OOD detection in practice [45,52,53]. However, MSP may perform poorly when ID and OOD data distributions are similar or when the ID data are significantly different from the training data used to train the classifier. In such cases, more sophisticated methods, such as MAH or energy-based models, may be more effective.

**Fig. 4.**
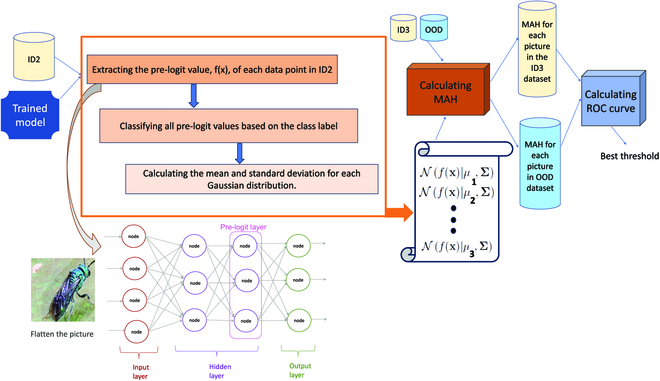
OOD detection process for MAH. Mahalanobis distance algorithm utilized trained insect classifier and validation ID data to option the approximate Gaussian distribution of the feature vector (here, the pre-logit) conditioned on each class. Then, the Mahalanobis distance of test ID and OOD data to the nearest class conditional normal distribution is calculated and a threshold is obtained. The pre-logit layer of neural network of an image is shown with a box around it.

**•** MAH-based algorithm [45] is another commonly used method for OOD detection in deep learning models. This OOD algorithm defines a confidence score based on an induced generative classifier of the potential labels according to the Gaussian discriminative analysis framework. The Mahalonobis distance algorithm follows the following steps (Fig. 4): First, it extracted the output of the penultimate layer of the trained insect classifier for each data (*x*, *y*), *y* = *c* in the validation set (ID2), denoted as *f*(*x*). Second, it assumed that all *f*(*x*) belonging to each class follow the multivariate Gaussian distribution. It then calculated the Gaussian distribution parameters (mean and variance) for each class. Mean and variance are denoted by *μ_c_* and $\hat{\Sigma }$, respectively. In our case, we had 142 classes of insects. Therefore, mean and variance for each of *c ∈ {1, 2, …, 142}* Gaussian distributions are calculated as follows: $\begin{array}{c} P\left(f\left(x\right)|\;y\;=\;c\right)\;=\;N\left(f(x)\;\;\;|\;\mu_{c}\;\;,\hat{\Sigma }\right) \end{array}$. Third, the confidence score for each input data, *M*(*x*), is defined as the Mahalonobis distance of pre-logit value, *f*(*x*), an unseen input *x* to the closest class-conditional Gaussian distribution as follows:

$$\begin{array}{c} M\left(x\right)=\underset{c}{\text{max}}-{\left(f\left(x\right)-{\hat{\mu }}_{c}\right)}^{T}{\hat{\Sigma }}^{-1}\left(f\left(x\right)-{\hat{\mu }}_{c}\right) \end{array}$$ (1)

MAH is the normalized distance, with the normalization performed using the covariance matrix of the training data. This helps account for correlations between features and results in a more accurate distance measure. Finally, similar to the MSP and EBM algorithms, the process of finding the threshold involves calculating the ROC curve for ID3 and OOD data and then selecting the threshold that achieves the most balance between FPR and true-positive rate (TPR).

**•** EBM [39]. The energy model is a function *E*(*x*) : ℝ*^D^* → ℝ, where each input value *x* is mapped to a non-probabilistic energy value *E*(*x*). These energy values can be transported into probability density utilizing the Gibbs distribution:

$$P\left(y|x\right)\;=\frac{e^{-E\left(x,y\right)/T}}{\int_{y'}e^{-E\left(x,y\;'\right)/T}}$$ (2)

There is a great similarity between the above formula and the softmax formula where *E(x, y)* is replaced by logit vector *f(x)*. In the above formula, the parameter *T* is called the temperature, which is set to *T* = 1. The Helmholtz free energy function *E(x)* for *x* ∈ ℝ*^D^* can be calculated from the denominator of the above formula as follows:

$$E(x)=-\;T\;\text{log}(\sum_{i}^{C}e^{f_{i}(x)/T})$$ (3)

The EBM operates based on the assumption that the energy value associated with ID data is notably lower than that of OOD data. In the process of inference, the energy score assigned to an unseen input is then compared to the energy scores of the training data. If the energy score of the input is significantly higher than the highest energy score of the training data, it is likely an OOD sample.

The form of the energy function is motivated by other EBMs like Boltzmann machines and variational autoencoders, where the energy function is related to the Gibbs distribution and the associated free energy. The OOD detection process of EBM is similar to MSP (Fig. 3), where the softmax value is replaced with the energy score.

### Insect classifiers methods

The architecture of a classifier plays a critical role in determining both the accuracy and efficiency of the model. Achieving the optimal design for a given task often requires careful consideration and experimentation. It is important to note that the primary focus of this paper is not on the classifiers themselves but rather on how their prediction performance is related (or not related) to OOD performance. This is addressed by utilizing three diverse types of architectures that have demonstrated significant success in practical image classification applications. These three network architecture choices, combined with the three OOD algorithms described in the previous subsection, offer a broad cross-section of approaches for evaluating OOD performance in the context of our specific insect classification problem.

We consider the following architectures:

**•** ResNet50: ResNet50, a variant of the convolutional neural network (CNN) model proposed by He et al. [18], has gained widespread popularity for its exceptional performance in computer vision and image classification tasks. The model owes its success to incorporating skip connections within its residual blocks, effectively addressing the challenge of diminishing or exploding gradients. Our implementation employs ResNet50, featuring 50 deep convolution layers.

**•** RegNetY32: RegNet is an optimized design space developed by Radosavovic et al. [54], where they explore a diverse set of parameters of a network architecture like width, depth, groups (commonly called as AnyNet, an initial space of unconstrained models that uses models like ResNet50 [18] as its base). By conducting numerous experiments with different parameter values for the design space, they successfully developed the optimized RegNetX. The combination of the well-known squeeze-and-excitation (SE) operation with ResNetX produced RegNetY, which exhibited outstanding performance [55]. We employed the RegNetY32 model in this study.

**•** VGG11: VGG is a CNN introduced by the Visual Geometry Group (VGG) at the University of Oxford [19]. This CNN architecture uses RELU activation function and pretrained weights (trained on ImageNet) to reduce computation load. We use VGG11, which is a variant of VGG. VGG11 is an architecture with 11 weighted layers. It includes nine convolutional layers and three fully connected layers.

### Datasets

We curated two series of datasets: one for ID and one for OOD. The ID data served three main purposes: (a) training and testing the insect classifier, (b) training the MAH algorithm, and (c) testing the performance of OOD algorithms in distinguishing ID data from OOD data. It is important to note that, given our use of AUROC and FPR95 (FPR at a 95% TPR) as performance measures, we eliminate the need to find the ID and OOD separation threshold. Therefore, no validation set for hyperparameter tuning is required. To achieve these objectives, we curated an insect dataset comprising 142 agriculturally relevant species with a significant economic impact on North American agriculture. This dataset is a subset of the publicly available iNaturalist dataset [56], augmented with seven classes of insects collected by the Department of Agronomy, Iowa State University, totaling 2 million insect images. The dataset was split into three separate sets for training the insect classifier, training the OOD algorithms (only for MAH), and testing the OOD algorithms, with a consecutive ratio of 70%, 15%, and 15%, respectively.

**Fig. 5.**
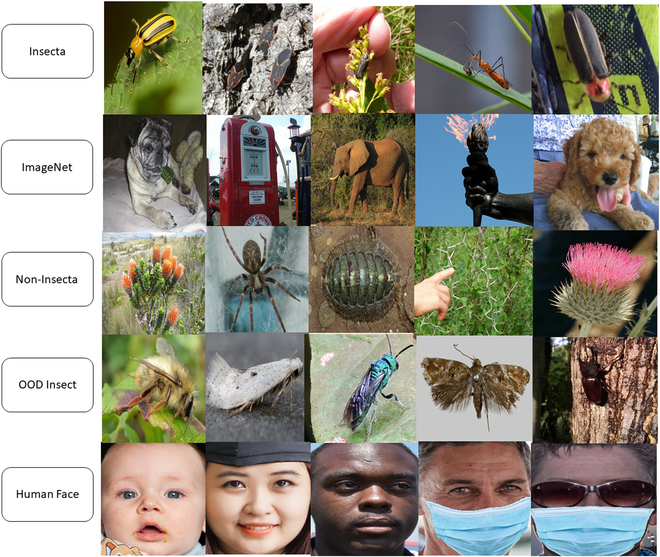
Dataset visualization. The Insecta dataset was used as ID datasets, and ImageNet, Non-Insecta, OODInsect, and Human Face were used as OOD datasets.

For OOD data, we utilized four datasets with different degrees of similarity to the ID insect data. An illustration for the datasets is provided in Fig. 5. We briefly describe each of these datasets.

• ImageNet [57] (far OOD): We start with the ImageNet 2012 classification dataset consisting of 1.2 million images across 1,000 object categories. We then took 10 random samples from each class and excluded all insect-related objects (e.g. bee, cricket, and ant) from it, which resulted in 9,730 samples.

• Human Face [58] (far OOD): We use the dataset from the face mask recognition dataset of the Kaggle competition (https://www.kaggle.com/datasets/ashwingupta3012/human-faces), which includes pictures of human faces with and without masks. The dataset consists of 3,059 images.

• Non-Insecta [56] (near OOD): This dataset is a subset of iNaturalist, where we exclude all the Insecta images from it. This dataset consists of 74,740 images.

• OODInsect (near OOD): This dataset includes all insect pictures that do not belong to any of the 142 classes of ID data. This dataset is also collected from the iNaturalist dataset. This dataset includes 56,487 pictures of OOD insects.

Taken together, these four datasets represent a diverse set of OOD images when compared to the dataset on which the insect classifier is trained.

### Metrics for OOD performance evaluation

We employed AUROC and FPR95 to evaluate the performance of OOD detection algorithms. We briefly discuss each metric below.

AUROC: AUROC [area under the receiver operating characteristic (ROC) curve] serves as a performance measure for classification tasks, specifically in distinguishing between ID and OOD. A higher AUROC indicates better OOD detection performance. This metric is particularly useful for OOD detection because it captures the overall performance of a model across various operating points. It is important to note that AUROC does not provide information about the specific operating point at which the model achieves its highest performance. Therefore, additional metrics such as FPR95 are employed to further evaluate OOD detection performance.

FPR95: FPR95 represents the probability that a negative example (OOD) is wrongly classified as positive (ID) when the model is at a high TPR, which is crucial for OOD detection. A lower FPR95 indicates that it is less likely to misclassify OOD samples as ID samples.

## Results and Discussion

We conducted a significant number of computational experiments along three performance axes: (a) classifier architectures and accuracies, detailed in the “Impact of classifier accuracy (and architecture) on OOD detection” section; (b) the degree of similarity between OOD dataset features and ID data, explained in the “Impact of degree of out-of-domainness on OOD detection” section; and (c) varying sample sizes of OOD data used to train the OOD algorithms, elaborated in the “Effect of data imbalance on OOD detection” section.

Here are the key findings: Our experiments indicate that the EBM outperforms the other two OOD detection algorithms (MAH and MSP). Additionally, our results from training classifiers to address the first hypothesis reveal that the RegNetY32 architecture exhibits superior performance in insect detection compared to the other two model architectures (ResNet50 and VGG11). Combining EBM with a RegNetY32 classifier results in an excellent insect classifier.

**Fig. 6.**
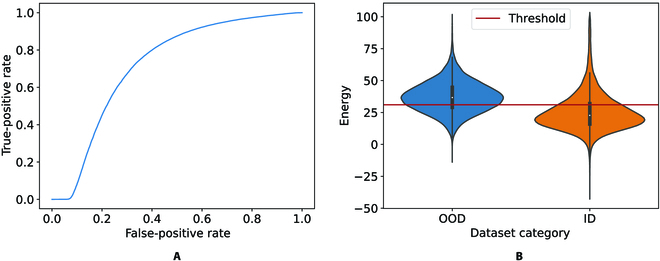
The performance evaluation of EBM on RegNetY32. (A) AUROC curve of binary classification between ID and OOD for the range of energy thresholds in the proposed EBM OOD detector. (B) Boxplot diagram of OOD and ID distribution.

We present the AUROC curve for this classifier in Fig. 6A, illustrating the aggregated OOD classifier performance across all possible energy level thresholds. Our analysis indicates an AUROC of 0.72. To optimize our results, we searched for the threshold that yielded the most promising outcome. Our analysis determined that a threshold of 31.0 provided the OOD classifier with the lowest FPR of 0.31 and the highest TPR of 0.69 (corresponding to FPR = 0 and TPR = 1). Setting the energy level to this threshold resulted in an accuracy of 68.97 for our OOD model.

In order to interpret the threshold and AUROC results, the energy distribution of ID and OOD data was further investigated.Figure 6B captures the boxplot illustration of OOD and ID data distributions. They exhibit the following interquantiles: ID interquantile of {min: −11.23, IQ1: 29.00, median: 36.68, IQ3: 44.71, max: 676.69}, and OOD interquantile of {min: −43.73, IQ1: 16.33, median: 23.69, IQ3: 34.30, max: 2,445.05}.

Majority of ID data were retained, while an acceptable portion of OOD was excluded when using an energy threshold level of 31.0. The wide scattering in the energy level of OOD data indicated the diversity of OOD images. Difficulty in OOD classification was also evident, as there was a partial overlap of low energy values on both ID and OOD.

### Impact of classifier accuracy (and architecture) on OOD detection

We explored the interplay between the accuracy of the insect classifier and the performance of the OOD algorithm. This question is motivated by a recent empirical observation [59] that suggests that improving the accuracy of the classifier is correlated with its ability to identify when an input does not belong to any of its classes. This observation is particularly appealing because one could invest effort in improving the accuracy of the classifier (via improved data collection, augmentation, hyperparameter tuning, and label smoothing) and get the added benefit of improved OOD detection.

**Fig. 7.**
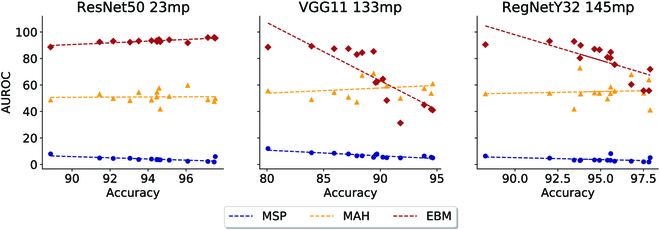
The trend of AUROC for the three OOD algorithms (MSP, MAH, and EBM) over the increment in insect classifier’s accuracy for the three architectures (ResNet50, RegNetY32, and VGG11). Each diagram specifies the performance of one architecture, and diagrams are sorted from left to right based on the increase in the number of weight parameters (*mp* stands for million parameters).

To explore this hypothesis, we created classifiers with a wide range of accuracies and compared the OOD algorithms’ performance on them. Specifically, we first created a sequence of 15 trained insect classifiers with increasing accuracy for each of the three types of network architectures (ResNet50, RegNetY32, and VGG11). These models are trained using cross-entropy loss function and AdamW optimizer with a learning rate of 1 × 10^−3^ and a batch size of 256. Their performance on the validation dataset ranged from an accuracy of 80% to 97.5% across these 45 models. Second, we ran all three OOD algorithms (MSP, MAH, and EBM) on these 45 trained insect classifiers. For evaluating the OOD performance, we consider the non-Insecta dataset as the OOD dataset. We ensured that both ID and OOD test datasets have the same size by random sampling of 74,740 (the size of the non-Insecta dataset) from ID data. The result of our analysis is shown in Fig. 7.

We observe several trends. First, the performance of the EBM algorithm is (almost always) better than the other two OOD algorithms across all three architectures. However, notice that improving the accuracy of the classifier does not improve the EBM-based OOD detection performance, especially for architectures with a very large number of trainable parameters; VGG11 has 133 million trainable parameters, and RegNetY32 has 145 million trainable parameters. For architecture with a moderate number of parameters (i.e., Resnet50 with 23 million trainable parameters), the observation of [59] holds. On the other hand, the other two OOD algorithms (MSP and MAH) show slight to no improvement in OOD performance as classifier accuracy is increased.

### Impact of degree of out-of-domainness on OOD detection

In this subsection, we explore how OOD performance is affected by the type of OOD datasets considered. This is particularly important to understand for agricultural applications, where the adoption of these tools may be compromised if the classifier makes obvious classification mistakes, for instance, classifying a human face as an insect.

We considered four datasets—Human Face, ImageNet, Non-Insecta, and OODInsect—that exhibit increasing contextual variations from the insect dataset used to train the classifiers. The non-Insecta and OODInsect datasets share many similar features to the in-domain dataset, such as leaves or trees in the image background; on the other hand, ImageNet and Human Face share less similar features to the ID insect dataset. These datasets are visualized in Fig. 5. Furthermore, to avoid potential bias from different sizes of datasets, we took a random sampling of 3,059 (our smallest OOD dataset size) from each of these datasets. We then measured the performance of the three OOD algorithms on our best-performing classifier.

**Fig. 8.**
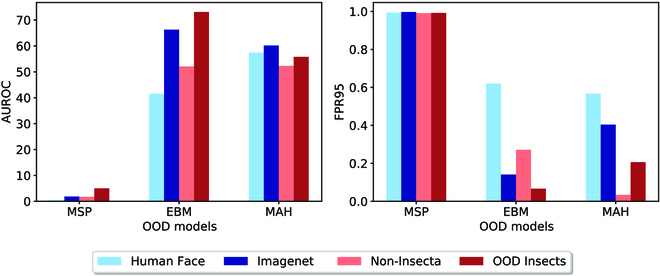
OOD detection algorithm performance across OOD datasets with different degrees of similarity to ID. Two metrics of AUROC and FPR95 are chosen to show two performance aspects. (Right) True Positive rate at False Positive rate 95%; the lower the FPR95, the better performance of OOD detector. ROC curves are provided in the GitHub repository mentioned in the data availability section.

Figure 8 lays out the results of this analysis. We observe that EBM has the best performance on all four OOD datasets—exhibiting consistently good AUROC, as well as low FPR95. MSP exhibited high FPR95, independent of the type of OOD data. This has been well documented [39]. EBM and MAH performed better than MSP on all OOD datasets. In particular, better performance was noted in the ImageNet dataset and OODInsect. This could be because the classifier is pretrained on Instagram images, which share similar features to the ImageNet dataset, and is trained on insect data that are similar to the OOD insect dataset. This makes the OOD detector more robust on these datasets and suggests that the similarity of features between the images the classifier is trained (or pretrained on) and the OOD samples can help improve the OOD detection. This observation suggests that using models pretrained on diverse/large datasets may produce especially robust OOD detection.

### Effect of data imbalance on OOD detection

Here, we explore how data imbalance in the OOD training data impacts OOD performance. We consider three subsets of ID data to train the OOD algorithms. We call these three datasets balanced, unbalanced, and unbalanced-uniform. For consistency, we ensured that each dataset had the same number of images (58,362 images). In the balanced subset, all classes have the same number of images (411 images per class). The unbalanced subset, on the other hand, exhibits the same data distribution as the original 2M image dataset used to train the classifier. Finally, in unbalanced-uniform, we considered a data distribution that is completely random, i.e., the size of each class is an independent uniform random variable. This allows us to explore the impact (or lack thereof) of specific data distributions exhibited by the unbalanced versus the unbalanced-uniform datasets. We compared these three ID subsets with the Non-Insecta OOD dataset on OOD detection performance on our best classifier.

**Fig. 9.**
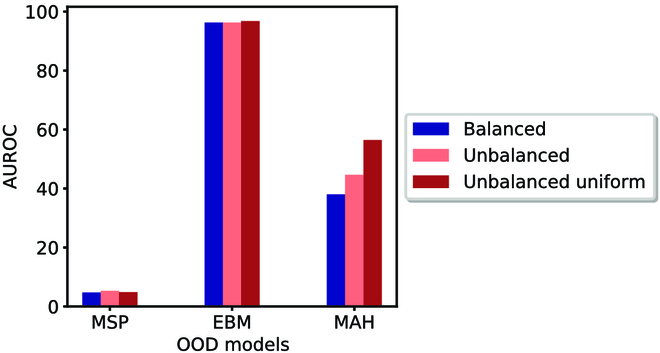
OOD performance across different levels of equilibrium of ID dataset. Only the MAH algorithm shows a significant difference in performance between these three subsets. ROC curves are provided in the GitHub repository mentioned in the data availability section.

The result of this analysis is shown in Fig. 9. EBM—a discriminative OOD algorithm—is robust to data imbalance and performs very well with high AUROC. On the other hand, the generative OOD algorithm, MAH, shows sensitivity to data imbalance. Overall, we observe that using EBM is particularly promising for agricultural applications, where there is often the possibility of data imbalance.

This analysis is especially important in agricultural applications, particularly for the insect classification problem, due to the possibility of significant data imbalance. For instance, charismatic species like the monarch butterfly are typically heavily imaged, while rare (or uninteresting) species can have very few samples in a dataset. One of the largest insect datasets, the iNaturalist dataset, consists of 13 million insect images. This dataset exhibits a large data imbalance between classes, with some classes containing fewer than 100 images and others containing more than 100,000 images. Such data imbalances are rather common in agricultural applications, and a clear understanding of the impact of such data imbalance on OOD performance is useful for practical deployment.

## Conclusions

Automated insect pest identification is an economically critical agricultural task. It is important that well-trained models, when deployed in the wild, abstain from making predictions when encountering data that are out of their training distribution. We explored and quantified the performance of several OOD approaches applied to insect pest classification. We used several insect classifiers that were trained on 2 million images to accurately classify 142 agriculturally relevant insect species. For this particular application, the EBM algorithm for OOD detection was the best-performing model. This work is the first step toward robustifying the performance of classifiers deployed for agricultural applications.

We conducted an extensive series of computational experiments to assess the performance of OOD algorithms, yielding valuable insights for the agricultural community. First, we observed a correlation between classifier performance and OOD detection accuracy in models with fewer parameters; however, this correlation diminished in more complex models. Second, the EBM OOD detector demonstrated superior performance when OOD datasets closely resembled the training and pretraining datasets. Third, empirical observations highlighted that discriminatively selected OOD detection algorithms, specifically EBM and MSP, exhibited insensitivity to data imbalance, in contrast to their generative counterpart algorithm, MAH.

The OOD approach outlined in this paper empowers the classifier to abstain from predictions when the input data diverge from the training distribution. This establishes a systematic mechanism for autonomous agricultural applications to solicit human intervention, thereby enhancing the reliability and trustworthiness of these tools. We foresee the potential application of this approach across various agriculturally significant classification tasks, including scouting and identifying biotic (disease) and abiotic stresses (nutrient deficiency), while also aiding in agricultural decision-making processes.

## Data Availability

We uploaded the source code for reproducing the analysis, and ROC curves associated with the results figures to the GitHub repository: https://github.com/mojdeh-saadati/iNat-OOD.git. OOD datasets: ImageNet (https://www.image-net.org/challenges/LSVRC/2012/), Human Face (https://www.kaggle.com/datasets/ashwingupta3012/human-faces), OODInsect (https://doi.org/10.5281/zenodo.7987921), and Non-Insecta (https://github.com/visipedia/inat_comp/tree/master/2017). ID dataset: The ID test data, ID3, along with the trained models, are accessible through the following link: https://doi.org/10.5281/zenodo.7987921.
